# Circulating Biomarkers for Laboratory Diagnostics of Atherosclerosis—Literature Review

**DOI:** 10.3390/diagnostics12123141

**Published:** 2022-12-13

**Authors:** Gabriela Bordeianu, Ivona Mitu, Raluca Stefania Stanescu, Corina Paraschiva Ciobanu, Elena Petrescu-Danila, Afrodita Doina Marculescu, Daniela Cristina Dimitriu

**Affiliations:** Department of Morpho-Functional Sciences II, University of Medicine and Pharmacy “Grigore T. Popa”, 700115 Iasi, Romania

**Keywords:** atherosclerosis, cardiovascular disease, ceramides, risk scores

## Abstract

Atherosclerosis is still considered a disease burden with long-term damaging processes towards the cardiovascular system. Evaluation of atherosclerotic stages requires the use of independent markers such as those already considered traditional, that remain the main therapeutic target for patients with atherosclerosis, together with emerging biomarkers. The challenge is finding models of predictive markers that are particularly tailored to detect and evaluate the evolution of incipient vascular lesions. Important advances have been made in this field, resulting in a more comprehensible and stronger linkage between the lipidic profile and the continuous inflammatory process. In this paper, we analysed the most recent data from the literature studying the molecular mechanisms of biomarkers and their involvement in the cascade of events that occur in the pathophysiology of atherosclerosis.

## 1. Introduction

Atherosclerosis is characterized by the deposit of a plaque composed mainly of lipids (atheroma) located on the wall of the arteries. Ultimately, these plaques can cause damage to the arterial wall (sclerosis), lead to obstruction of the vessel, or even rupture, with often dramatic consequences. Within atheroma plaques, inflammatory cells and lipids reorganize, along with other elements, leading to a local modification of the appearance and structure of the wall. Blood cells can secondarily associate with these plaques. Their thickening or rupture is responsible for potentially severe, even fatal, clinical manifestations (myocardial infarction, stroke).

The research regarding the complex mechanisms involved in the process of atherosclerosis constantly brings information about potential biomarkers, overexpression of certain genes and vascular processes that occur in the clotting cascade. Nowadays, the challenge is represented by identification of new quantifiable markers that can provide useful information for the diagnosis, monitoring or screening of atherosclerotic disease, but with an advantageous cost–benefit ratio.

Carrying out an evaluation analysis of established markers versus new markers in order to improve the laboratory diagnosis of atherosclerotic disease is a challenging task. The formation of atheroma plaques is the consequence of a constellation of factors, including an excess of circulating lipids in the blood, inflammatory processes, and alteration of endothelial integrity. Due to these complex mechanisms, there are still no ideal markers established, and integrated analysis and interpretation of the obtained data in the clinical context are necessary.

In the current review, we aim to thoroughly describe circulating lipid markers, inflammatory markers, and parameters that assess the thrombotic status, which are useful for evaluating the stage of the atherosclerotic process.

## 2. Lipid and Lipoprotein Profile Markers

Atherogenic dyslipidaemia is characterized by a high level of LDL-c (low-density lipoprotein cholesterol), accompanied by an increase in TG (triglycerides), an increased number of sdLDL (small dense LDL), low HDL-c (high-density lipoprotein cholesterol), and postprandial hyperlipidaemia [1,2,3]. Atherogenic dyslipidaemia is considered to be responsible for the development and progression of the atherosclerotic process. The panel of atherogenic risk markers consists of non-HDL-cholesterol and apolipoprotein B-100 measurements, together with LDL-c, which measures the free and esterified cholesterol in LDL [4].

Along with the determination of lipoprotein concentration in the blood, it is important to take into consideration that the lipoprotein classes encompass particles with distinct protein and lipid profiles. In addition to the traditional risk factors, such as those established by Framingham study, new biomarkers emerged [2].

### 2.1. Low-Density Lipoprotein (LDL) and Oxidized Low-Density Lipoprotein (ox-LDL)

Many studies have demonstrated that high levels of LDL represent a risk factor for cardiovascular diseases associated with atherosclerosis [5]. The structure of LDL particles is represented by a hydrophobic core (built up by cholesterol esters and triglycerides) surrounded by amphipathic lipids (free cholesterol and phospholipids). Apolipoprotein B-100 is also located on the surface of the particle, with an estimated 90% of the total Apo B-100 in circulation belonging to LDL alone. LDL is formed from the catabolism of VLDL (very low-density lipoproteins), which includes the formation of IDL (intermediate-density lipoprotein). VLDL and IDL also contain Apo B-100 [6].

The role of LDLs is to transport the hydrophobic cholesterol in the aqueous medium of blood plasma [3,4,7]. Afterwards, the LDL particles are transferred into the cells by receptor-mediated endocytosis, thus providing the necessary cholesterol [8].

LDL is considered the most important factor involved in the process of atherosclerosis. When oxidative stress is present, an excessive amount of reactive oxygen species is generated, and LDL particles are oxidized (ox-LDL). It is considered that ox-LDL, rather than native LDL has atherogenic traits [7]. Additionally, a high concentration of ox-LDL in atherosclerotic plaques makes them more prone to rupture [5]. Morphological modifications that occur at the level of endothelial cells lead to an increased permeability for LDL particles. The accumulation of ox-LDL in the subendothelial space is one of the initial events in the formation of atherosclerotic plaque [7].

Since the link between LDL and atherosclerosis was highly studied, beginning in the 1950s [4], the determination of LDL-c has become a routine in most laboratories. The Friedewald formula is used to calculate LDL-c, using the concentrations of total cholesterol (TC), HDL-cholesterol (HDL-c), and triglycerides: LDL-c = TC − HDL-c − TG/5. The results are expressed in mg/dL [4,9]. Factors such as high levels of triglycerides (>4.5 mmol/L) or low levels of LDL-c (<1.8 mmol/L) in the sample lead to an underestimation of LDL-c [3]. Direct assays of LDL-c are also available, although these may lead to inaccurate results in the case of the structural modification of lipoprotein particles [4]. For research purposes, the determination of ox-LDL was performed using autoantibodies, murine monoclonal antibodies, or malondialdehyde -LDL, but the assays did not prove to be robust [10,11]. The quantity of cholesterol in LDL particles may vary, but there is just one ApoB protein per atherogenic particle (VLDL, IDL, LDL). ApoB represents a better estimation of the number of LDL particles in circulation, compared to LDL-cholesterol [12].

### 2.2. Small-Dense Low-Density Lipoprotein (sdLDL)

A novel marker of atherosclerosis is sdLDL, a subset of LDL, which is probably the best studied marker of this disease. The LDL class of lipoproteins is represented by particles which are heterogenous in size and density, while sdLDL is characterized by a diameter smaller than 25.5 nm and density higher than 1034 kg/L. These sdLDLs are considered the most atherogenic particles among the subtypes of LDL class [4,13,14]. Hypertriglyceridaemia favours the formation of sdLDL. The VLDL rich in triglycerides are metabolized by the action of lipoprotein lipase. The cholesterol-ester transfer protein transports triglycerides from VLDL to HDL particles, and cholesterol-esters from LDL and HDL to VLDL particles, leading to an enrichment in triglycerides in VLDL. The hepatic lipase will act upon those particles, transforming them into smaller and denser molecules [13,14].

Compared to LDL, sdLDL has a lower affinity for the LDL receptor, which leads to a prolonged circulation time and is more susceptible to alterations (oxidation, glycosylation). Their small size allows them to penetrate easily in the subendothelial space, where they are preferentially uptaken by macrophages, thus contributing to the process of atherosclerosis [13,14,15].

A novel generation of tests that measure the number of LDL and sdLDL particles, together with the determination of triglycerides and sphingomyelin content of LDL, may provide a better estimation of the cardiovascular risk [4].

### 2.3. High-Density Lipoprotein Lipidome

The concept that high levels of HDL-c represent a protective factor against atherosclerosis is being challenged. Therapeutical interventions that led to an increased level of HDL did not reduce the cardiovascular risk. Attention is now drawn towards the ability of HDL to promote the cholesterol efflux from macrophages, which seems to be inversely correlated with atherosclerosis risk [16,17] and also towards other functions of HDL, including involvement in the process of inflammation, antioxidative properties, and antidiabetic effects. Since HDL particle components are very heterogenous in size, surface charge, and composition, new therapeutic strategies focus on improving the function of HDL, rather than increasing the level of HDL-cholesterol [18].

An emerging technology, proton nuclear magnetic resonance (^1^H-NMR), is used in lipidomic assessments. The technique provides a valuable insight on the identification and quantification of lipid components [19]. A study investigated the lipid profile (HDL and non-HDL) using the proton NMR-based lipidomic analysis, in 99 patients with coronary heart disease. The alteration in lipid profile, exposed by this technique, allowed a distinction between the stages (mild, moderate, and severe) of coronary stenosis [20]. The same technique was used in another study to evaluate the same two profiles in patients with triple vessel disease, pathology defined as “a ≥50% diameter luminal narrowing in all 3 major epicardial vessel systems“. Patients with coronary heart disease had lower levels of phospholipids and polyunsaturated fatty acids, and higher levels of saturated fatty acids, cholesterol, and triglycerides [21].

Kostara et al. investigated the HDL composition in healthy patients compared to patients with a recent diagnosis of type 2 diabetes mellitus and patients with acute coronary syndrome. They reported a progressive alteration of HDL composition within these groups, reflected in high levels of triglycerides, lysophosphatidylcholine, and saturated fatty acids, accompanied by low cholesterol, phosphatidylcholine, phosphatidylethanolamine, sphingomyelin, plasmalogens, and polyunsaturated fatty acids [19]. Another study confirmed that coronary heart disease and type 2 diabetes mellitus patients present an alteration of composition and function of HDL [22]. For patients with diabetes, an increase in small triglyceride-rich particles was reported in the HDL composition, in detriment of large and very large particles, an alteration that could be due to an increased CETP (cholesteryl ester transfer protein) activity. The situation is reversed in patients with coronary heart disease, with an increase in large HDL particles.

Although determination of HDL-c is used extensively in medical practice, new studies suggest that it should be replaced by biomarkers that could better evaluate the correlation of HDL-c level with the atherosclerosis process [4].

Monocyte-to-high-density lipoprotein ratio (MHR), a novel marker of inflammation, is considered to have prognostic value regarding cardiovascular diseases and mortality [23]. An increased ratio was associated with a poor outcome at three months after an acute ischemic stroke in patients with atherosclerosis [24].

Another determination that can estimate the atherogenic risk is represented by the calculation of the so-called “non-HDL cholesterol”: non-HDL-c = TC − HDL-c. The advantage of this determination is that it takes into consideration the cholesterol in the remnant lipoproteins (remnant chylomicrons, remnant VLDL) and in lipoprotein (a) (Lp (a)). The use of non-HDL-c was proposed for patients with high levels of triglycerides that would not allow an accurate estimation of LDL-c, calculated with Friedewald equation. Several studies showed that non-HDL-c is as good as LDL-c in evaluating the risk for cardiovascular diseases (or even better in the presence of hypertriglyceridaemia) [3] and, moreover, non-HDL-c is used for the estimation of 10-year fatal and non-fatal cardiovascular disease risk in SCORE2 («Systematic COronary Risk Evaluation», age 40–69 years) and SCORE2-OP («Older Persons», age 70–89 years) algorithms, developed by the European Society of Cardiology [25].

ApoA1 is the major protein in HDL particles. The ratio ApoB/ApoA1 is considered an indicator of the balance between pro- and antiatherogenic lipoproteins, with a high ratio being associated with a high risk [6]. A study conducted by Florwall et al. showed a strong association between ApoA1 and cardiovascular morbidity and mortality in a group of Swedish men, 77 years of age, who were selected from the participants in the Uppsala Longitudinal Study of Adult Men (ULSAM) [26].

Patients treated with statins are considered to be at risk for atherosclerotic cardiovascular disease, even in the presence of optimal levels of LDL cholesterol. Statins inhibit the synthesis of cholesterol, but triglyceride-rich lipoproteins also have atherogenic potential. A recent study with a set of participants treated with statins, selected from the Copenhagen General Population Study, demonstrated that an increased risk was correlated with high levels of ApoB and non-HDL cholesterol concentrations, not with high LDL cholesterol concentrations [27].

### 2.4. Triglycerides (TG)

Triglycerides travel in plasma as a component of chylomicrons (CM), very low-density lipoprotein (VLDL) and their remnant particles. Chylomicrons contain apolipoprotein ApoB-48 and triglycerides from dietary fat, while VLDL particles contain Apo B-100 and triglycerides synthesized in the liver. Lipoprotein lipase, located on the vascular endothelial surface, degrades the circulating triglycerides [28]. Fatty acids released from triglycerides lead to dysfunction of the vascular endothelium, one of the first traits of the atherosclerotic process and increased oxidative stress [29,30]. Apo CIII, an apolipoprotein mostly associated with VLDL, may inhibit the activity of lipoprotein lipase and may decrease the uptake of particles which contain triglycerides by the tissues. A recent study demonstrated that apolipoprotein CIII is associated with the risk of cardiovascular events in patients with coronary artery disease [31].

Hypertriglyceridaemia (HTG), defined as a serum triglycerides level >150 mg/dl, is strongly associated with cardiovascular disease. A study conducted on a population of Korean adults, aged 30–49 years, showed that hypertriglyceridaemia is an independent risk factor for cardiovascular events [32], while another study, on the Italian population, confirmed the association between hypertriglyceridaemia and an increased risk of atherosclerotic cardiovascular events [33].

A recent study, which selected patients from two observational cohort studies, demonstrated the usefulness of measuring the average triglycerides levels over time instead of taking into consideration one single measurement or the maximal value. Additionally, the study reported an association between TG and cardiovascular risk even at a level of TG between 100 and 150 mg/dL [34].

### 2.5. Lipoprotein (a)

Lipoprotein (a) is a type of lipoprotein considered to have a proatherogenic function at high concentrations. Lp (a) represents an LDL in which a molecule of apolipoprotein (a) (Apo (a)), a highly glycosylated protein, is attached covalently by one disulfide bridge to Apo B-100 [35]. Apo (a) presents a high level of homology with plasminogen, and it is an apolipoprotein with a high heterogeneity, both in size and in glycosylation. The heterogeneity in size has an impact on the measurement of Lp (a). The method based on latex-enhanced turbidimetry (kit assay by Denka Seiken, Japan) is considered very reliable [36]. Due to its homology to plasminogen, Lp (a) may interfere with thrombosis/fibrinolysis [2,10]. Lp (a) is synthesized in the liver, and the concentration in the general population varies between 0.1 mg/dL and 200 mg/dL. Since Lp (a) tends to accumulate at the site of injuries, it is supposed to be physiologically involved in tissue healing. As a carrier of oxidized phospholipids, high levels of Lp (a) have been linked to an increased cardiovascular risk [37].

A large, randomized study demonstrated the association between myocardial infarction and genetically elevated levels of Lp (a) [38]. It was also mentioned that the level of Lp (a) is a good predictor of coronary artery disease (CAD) risk in the absence of premature family history of CAD, and there are current studies that are focused on targeted therapies in reducing Lp (a) level [39].

### 2.6. Ceramides

Ceramides are bioactive lipids that belong to the sphingolipid family, being composed of a sphingosine backbone and a fatty acyl chain of varying length and degree of saturation [40]. They are synthesized by six different ceramide synthases and represent the scaffold for complex sphingolipids, e.g., sphingomyelin and the glycosphingolipids [41]. Ceramides perform important biological roles, being implicated in the signalling pathways that are associated with various cellular processes, such as cell growth, differentiation, and apoptosis [42]. They have also been shown to be involved in the pathophysiological mechanisms of a large array of human diseases, including atherosclerosis and cardiovascular disease (CVD), insulin resistance and type 2 diabetes mellitus, neurodegenerative disorders, etc. [43,44].

In circulation, ceramides are found incorporated in the plasma lipoprotein particles [45] (their concentration being higher in LDL compared with HDL or VLDL), and have been shown to accumulate in atherosclerotic plaques [40,46]. Ceramides are involved in the development and progression of the atherosclerotic process through several mechanisms. They stimulate the transport of oxidized LDL across arterial endothelial cells (transcytosis), promote the retention of LDL in atherosclerotic lesions, and enhance the uptake of LDL by macrophages [47,48,49]. Ceramides are produced in higher amounts in vascular endothelial cells in patients with atherosclerosis, and they lead to decreased NO (nitric oxide) production and the impairment of endothelium-derived vasorelaxation, thus contributing to the arterial dysfunction that is associated with CVD [50]. Ceramides are also involved in the chronic inflammation that is associated with the atherosclerotic process by promoting the production of C-reactive protein (CRP) and interleukin-6 (IL-6) [42].

#### Ceramides as Biomarkers for the Atherosclerotic Cardiovascular Disease

A first connection between ceramide and CVD was ascertained almost two decades ago by a study demonstrating that decreasing ceramide levels with an inhibitor of the de novo synthesis pathway prevented the development of atherosclerotic lesions in ApoE-deficient mice [51]. Since then, numerous studies using pharmacological agents and transgenic mice explored the role of ceramides in CVD.

Although the plasma levels of LDL-c and HDL-c are the most common markers associated with atherosclerosis, coronary heart disease (CHD) may be present in patients having these markers within the normal range [52]. Recent studies with large clinical cohorts, mostly performed in the past decade, revealed that circulating ceramide levels represent novel reliable biomarkers for the prediction of atherosclerosis and CV risk in the general population. Several pieces of evidence support this idea, as mentioned below. Ceramide levels have been found to be altered in the plasma prior to the appearance of atherosclerosis and CHD, having the potential to be used as subclinical markers for these conditions [52,53]. Ceramide concentrations in plasma have been shown to predict atherosclerotic plaque instability, being associated with the vulnerable plaque characteristics (the fraction of necrotic core tissue and lipid core burden) [54]. Increased levels of specific plasma ceramides have been shown to be associated with a greater severity of the coronary artery stenosis [55,56]. The concentrations of specific ceramide species have been found elevated in patients with STEMI (ST-segment elevation myocardial infarction), demonstrating a positive and independent association between the plasma levels of these ceramide types and the presence of plaque rupture [57]. Elevated plasma levels of ceramides have been demonstrated to be independently associated with major adverse cardiovascular events, including acute myocardial infarction and death, in patients with CHD [53,58].

The combined techniques of ultra high-performance chromatography (UHPLC) and mass spectrometry allow the measurement of plasma concentrations of ceramides with distinct N-acyl chain lengths and degrees of saturation. Among the many ceramide species that are present in circulation and that have been measured in clinical studies, three have been constantly associated with atherosclerosis and CHD, having higher values in patients compared to controls: Cer (16:0), Cer (18:0), and Cer (24:1) (all these are derivatives of C18-sphingosine) [53,59].

Since panels of single ceramide species are rather difficult to use in clinical practice, *ceramide risk scores* (Table 1) have been recently developed based on specific ceramide concentrations and their ratios, and they are currently used clinically for efficient risk stratification in the primary and secondary prevention of atherosclerotic CVD. The *CERT1* (ceramide test 1) score was developed by Zora Biosciences [53], being in operation in Finland and at the Mayo Clinic in the USA. It comprises six components: the concentrations of the three ceramide species previously mentioned—namely, Cer (16:0), Cer (18:0), and Cer (24:1)—and their ratios to Cer (24:0), a benign ceramide having no correlation with disease (it was found at lower concentrations in patients with CHD than in healthy controls, being considered a potentially cardio-protective ceramide species) [48]. According to this risk score, patients were stratified into four risk categories (low, moderate, increased, and high); the CVD risk increased linearly along with the increasing CERT1 score in patients with stable CHD as well as in patients with ACS. The comparison between the high- and low-risk categories showed a 4.2- and 6.0-fold increase of the relative risk in patients with stable CHD and ACS, respectively [60]. CERT1 predicted death in patients with CHD more than three times better than LDL-cholesterol, thus showing an improved performance in comparison with traditional biomarkers of CV risk [45].

*CERT2* score was subsequently developed by the same group by combining three phosphatidylcholine (PC) species with the same four types of ceramides used in CERT1, taking into account that PC levels have been shown to predict CV events. CERT2 was validated in subsequent large cohort studies and showed improved performance in stratifying CHD patients for their risk of developing CV events, in particular CV death [61]. CERT2 was significantly related with the levels of lipid biomarkers (LDL-cholesterol and triglyceride), inflammatory markers (hs-CRP and IL-6) and markers of myocardial necrosis and dysfunction (hs-TnT—high-sensitivity troponin T, and NT-proBNP—N-terminal probrain natriuretic peptide) [60].

Because of the strong correlations that have been found between the plasma levels of specific ceramides and CVD, that frequently have shown superior performance compared with the conventional CV biomarkers, serum ceramide levels have emerged as strong predictive biomarkers of CHD, major cardiac events, and death [47]. Specific plasma ceramides and the ceramide risk scores can allow the identification of CHD patients at high CV risk, and these patients may then benefit from specific management that extends beyond the standard care procedures.

Diagnostic tests that measure circulating ceramides are now available in some clinics in the United States and Europe [45,62]. Nevertheless, these biomarkers have not yet been included in standard practice since they can currently be performed in only a limited number of clinical laboratories worldwide [40].

## 3. Inflammatory Profile Markers

### 3.1. C-Reactive Protein (CRP), High-Sensitivity C-Reactive Protein (hs-CRP)

Numerous clinical studies associate the inflammatory process and atherosclerosis. It is still unclear whether inflammation is a cause or an effect of the process of atherosclerosis. Among the inflammatory markers, C-reactive protein (CRP) measured with high sensitivity methods (hs-CRP) has been studied to evaluate groups of patients with atherosclerotic risk [63]. CRP belongs to the group of proteins called pentraxins. These are multimer proteins and exist in different protein sizes. CRP is a short pentraxin, a pentameric protein synthesized by the liver, whereas Pentraxin 3 (PTX3) is a long protein, secreted by macrophages and endothelial cells. Both seem to be useful cardiovascular risk markers [64].

The main inductors of the CRP gene are IL-6, IL-17 [59], IL-1β, and tumour necrosis factor (TNF) [65]. CRP is involved in all stages of atherosclerosis by participating in various mechanisms such as complement activation, lipid accumulation, thrombosis, and monocyte recruitment. On the one hand, CRP activates the classical pathway with a proinflammatory function [66], and on the other hand, it can form a complex with the H factor (CRP-fH), thus preventing the formation of membrane attack complex (MAC) [67].

CRP induces a prothrombotic status either by (1) increasing the procoagulant activity, explained by the high expression of tissue factor at the level of monocytes, a phenomenon that occurs in the presence of B, T, and NK lymphocytes [68] or by (2) reducing fibrinolysis, explained by the high expression of PAI-1, the main inhibitor of fibrinolysis [69].

Hs-CRP is extensively used as a low-grade inflammation biomarker, mainly to assess cardiovascular risk. There is copious data supporting the association between high hs-CRP levels and atherosclerosis severity—expressed either as mortality and risk or as surrogate markers such as triglyceride concentration or body mass index [70,71,72].

Hs-CRP levels are usually measured using a latex particle-enhanced immunoturbidimetric assay; the measuring range for this assay is 0.01–20 mg/L [71]—it should be noted that the threshold values for cardiovascular risk stratification are: low risk at <1 mg/L, average risk at 1–3 mg/L, and high risk at >3 mg/L [70,73].

### 3.2. Pentraxin 3 (PTX3)

PTX3 is an acute phase protein with a modulatory function on the complement system, inflammatory response, angiogenesis, and vascular and cardiac remodelling [74]. Even though CRP, which is the prototype of the pentraxin family [75], together with PTX3 are both considered valid candidates for atherosclerosis, studies to date reported a lack of correlation between their serum levels, suggesting different mediation pathways of the inflammatory process in atherosclerotic plaques. Levels of PTX3 are measured using a high-sensitivity enzyme-linked immunosorbent assay system for human plasma: the normal concentration of plasma PTX3 is considered ∼2 ng/mL [76].

PTX3 is proven to be an independent prognostic factor for cardiovascular risk, independent of CRP levels [77], with studies reporting high plasma levels of PTX3 in patients with myocardial infarction or carotid stenosis. PTX3 also assesses plaque vulnerability [78] and is a reliable marker for risk stratification in patients undergoing percutaneous coronary intervention (PCI). Before PCI, the levels of PTX3 were associated with larger plaque area and volume and a higher risk of plaque rupture [79].

PTX3 is a potential biomarker of early endothelial dysfunction, since high levels are reported to decrease the production of nitric oxide through the upregulation of matrix metalloproteinase-1 and P-selectin [80,81]. In contrast, PTX3 suppression improves endothelial function [80,82,83]. Concerning the pathophysiological role of PTX3 in atherosclerosis, studies performed on PTX3 knockout mice reported vascular wall inflammation and a high uptake by macrophages of oxidized low-density lipoprotein cholesterol in atherosclerotic plaques, since PTX3 is normally located at the membrane of late apoptotic macrophages and mediates the phagocytosis of macrophages. Therefore, we can consider PTX3 as an antiatherogenic molecule [84]. There are studies that report a positive correlation between PTX3 and adiponectin or HDL-cholesterol, but no correlation with body weight, BMI, or fasting plasma glucose [85,86], suggesting that dysfunctional adipose tissue and metabolic syndrome status should be taken into consideration when interpreting PTX3 levels.

In inflammatory pathologies, PTX3 rises faster than CRP, with a peak at 6–8 h vs. 24–48 h for CRP, suggesting a possible clinical usage as complementary biomarkers. A valid explication for the different peaks could be the site for synthesis, which is local by a wide range of stromal or myeloid cells for PTX3 vs. systemic by hepatocytes for CRP [87].

In conclusion, PTX3 presents important implications in cardiovascular diseases, with a balance between its beneficial and negative effects that is challenging to maintain. On one side, PTX3 reduces inflammation, reduces macrophages infiltration, lowers the myocardial necrosis, and decreases the risk of coronary restenosis, and on the other side, it induces morphological alterations, increases blood pressure levels, increases the prevalence of coronary artery disease, and increases fibroblast growth factor 2 and tissue factor expression [88]. As an acute phase reactant, PTX3 is considered a biomarker of disease severity and outcome [89,90]. A recent study showed a correlation between high levels of PTX3 and the severity of coronary artery disease. Moreover, there is a positive correlation between PCSK9 (proprotein convertase subtilisin/kexin type 9), a regulator of hepatocyte LDL receptor, and PTX3, confirming once again its clinical use in assessing atherosclerosis [91].

### 3.3. Matrix Metalloproteinases (MMPs)

MMPs are a family of zinc-dependant endopeptidases involved not only in the processes of extracellular matrix degeneration or remodelling, angiogenesis, but also in atherosclerosis and fibrotic disease [92,93]. There are, in total, 28 members, of which 23 are expressed in human tissue and 14 are expressed in veins and arteries [94]. MMPs have beneficial effects such as maintaining vein wall structure and function, and also deleterious effects such as atherosclerotic plaque formation and instability [95,96]. Recent progress promotes the translation into clinical practice of MMPs as biomarkers for cardiovascular diseases.

A recent review reported that epigenetic mechanisms such as DNA methylation or histone acetylation/methylation modify the expression of MMPs, the transcription factors (that regulate their expression), and the tissue inhibitors of MMPs. These processes may be induced by medication (tetracyclines, statins), high prevalent diseases, and bacterial/viral infections, including SARS-CoV-2, and can significantly influence the progression and development of cardiovascular diseases [97]. One representative study presented a novel mechanism in atherosclerosis, describing how oxidized LDL upregulates microRNA-29b and leads to epigenetic modifications of MMP-2/MMP-9 genes. The consequence is a high primary human aortic smooth muscle cell migration [98].

Another novel mechanism is described in very early phases of atherosclerosis in mice. Platelets expressing MMP-2 interact with protease-activated receptor-1 (PAR-1) of endothelial cells and trigger the exposure of adhesion molecules, which in turn facilitate the adhesion and transmigration of monocytes through the endothelial monolayer and generate plaque development [99]. Furthermore, MMP-2 knockdown attenuates age-dependent carotid stiffness by increasing endothelial nitric oxide synthase levels and elastin to collagen ratio [100].

MMP-14 or membrane type-I matrix metalloproteinase (MT1-MMP) regulates the development of atherosclerosis, by promoting the cleavage of LDL receptor [101]. Moreover, genome-wide association studies found that MMP-12 correlates with all ischemic strokes, MMP-1 and MMP-12 with subtypes of stroke, large-artery atherosclerosis, and MMP-8 with small vessel occlusion [102].

The role of MMPs as biomarkers for cardiovascular disease is especially important in more advanced stages of the disease since their high activity increases the risk of plaque rupture [103,104]. Evidence has shown that in newly diagnosed patients with unstable angina pectoris, a specific MMP (MMP-9) can differentiate between those with plaque and those without plaque [105]. In addition, in patients with CHD, increased levels of MMP-9 at baseline were associated with cardiovascular death [104] and the severity of acute coronary syndrome [106]. Therefore, certain MMPs can help in predicting the stage of the disease and, moreover, introduce individualised therapy in secondary prevention.

## 4. Non-Specific Inflammatory Profile Markers

### 4.1. Fibrinogen

Fibrinogen is a glycoprotein synthesized by the liver and plays an essential role in the coagulation process. During vascular lesion, fibrinogen is subject to the action of thrombin and cleaved to fibrin. Fibrin is the most abundant component of the clot [107]. Numerous studies have associated increased levels of fibrinogen with vascular diseases by stimulating fibrin formation, together with increasing blood viscosity and platelet–platelet interaction [108,109]. On the other hand, fibrinogen has been shown to stimulate platelet aggregation and contraction of the injured cellular wall [110], while also contributing to cell proliferation [111] and participating in the regulation of cellular adhesion [112].

There are many studies that link fibrinogen level to coronary artery disease. Some epidemiological data show an association between fatal and non-fatal myocardial infarction and fibrinogen levels independent of other risk factors [113]. Other studies reported that fibrinogen level is an independent risk factor for sudden death and myocardial infarct (MI), mainly in patients with pre-existing coronary artery disease [114,115] and among people without known cardiovascular disease [116]. Even if a high level of fibrinogen is associated with increased cardiovascular risk, some studies that used drugs that lower fibrinogen level (fibrates) failed to show any beneficial effects [115,117]. Therefore, fibrinogen level is predictive of coronary artery disease, but it is still unclear whether fibrinogen represents a causal factor.

A study on 2288 Chinese patients who underwent coronary angiography for angina pain demonstrated that the severity and prevalence of coronary atherosclerosis correlated with fibrinogen values. In this study, the Clauss method was used for the quantitative measurement of plasma fibrinogen. The analysis of the ROC curve established a cut-off point of 3.21 g/L for predicting the severity of coronary stenosis [118]. These results are comparable to those obtained in a study conducted in Italy on 2121 patients evaluated by coronary angiography to establish the extent of coronary artery disease [119].

The Copenhagen City Heart Study studied the connection between the level of fibrinogen and ischemic stroke developed during 6 years of follow-up. The results showed an average value of 3.6 g/L in the group of patients with myocardial infarction; in the case of patients with advanced atherosclerosis (carotid artery stenosis >50%), an average value of 4.7 g/L versus 3.1 g/L in the control group was reported [120].

Some data indicate a high content of fibrin (60%) in intracoronary thrombi collected during thrombectomy in acute STEMI [121]. This associates with a denser fibrin network and a fibrin biofilm on the surface of intracoronary thrombi from acute myocardial infarction (MI) patients. This modified fibrin clot is less susceptible to lysis and was reported in patients with MI [122,123].

The prothrombotic fibrin clot is promoted by increased thrombin generation, platelet activation, and platelet-derived factors such as P-selectin associated with clot content [124].

Fibrin is involved in all stages of atherosclerotic lesion. The presence of microthrombus on the normal intima causes endothelial dysfunction with intimal oedema, an early characteristic of the atherosclerotic phenomenon. The presence of fibrin in intima contributes to the migration and proliferation of smooth muscle cells (SMC) causing the growth of atheroma plaque. On the other hand, the fibrinogen and fibrin degradation products (FDP) are mitogenic factors for SMC and act as chemoattractant for leukocytes. Fibrin in fibrous plaque seems to bind the lipoprotein Lp (a), immobilizing lipid structures at the level of the atherosclerotic lesion [125,126].

The central fibrinolytic enzyme is plasmin, which is derived from an inactive plasma precursor known as plasminogen. The activation of the plasminogen into plasmin takes place with the help of two serin proteases: tissue plasminogen activator (t-PA) and urokinase plasminogen activator (u-PA) [127].

Plasmin digests the fibrin clot resulting FDP and D-dimers, that are released into circulation [128].

### 4.2. D-dimers

D-dimers result from cross-linked fibrin degradation and are frequently used in clinical settings. Although D-dimer levels are mainly linked to embolic events, there are some studies supporting their use as a strong predictor of cardiovascular events and a potentially useful marker for the severity of coronary artery disease and subsequent major clinical events prediction [129,130]. The link between D-dimer levels and atherosclerosis is not completely elucidated—inflammation following atheroma formations and fibrinolysis may partially explain the physiopathological link—as empiric data is necessary to substantiate such a mechanism.

Besides the soluble fibrin degradation production, the action of plasmin exposes new carboxy-terminal (C-terminal) lysine at the fibrin surface. The presence of the lysine promotes the binding of plasminogen and, therefore, the transformation into plasmin and the binding of plasmin, conferring protection against α2-antiplasmin [131]. To prevent hyperfibrinolysis, there are some natural factors that negatively modulate the plasmin. Plasminogen activator inhibitor-1 (PAI-1) inhibits the action of t-PA and u-PA; α2-antiplasmin is a covalent inhibitor of plasmin; thrombin activatable fibrinolysis inhibitor (TAFI), a member of the metallocarboxypeptidase family, removes the C-terminal lysine from a partially degraded fibrin clot cancelling the fibrin function in the mechanism of fibrinolysis [131,132,133].

The plasminogen activator (PA) system may participate in the pathogenesis of atherosclerosis by modulating the turnover of intimal fibrin and extracellular matrix deposits and by contributing to intimal cell migration. This system generates plasmin that affects the fibrin degradation and the turnover of extracellular matrix deposits after metalloproteinase activation. On the other hand, plasmin activates cytokines that participate in the atherogenic mechanism [38,134,135].

Some investigators have reported that high PAI-1 and/or t-PA levels are associated with the presence of coronary artery disease. It was observed that there were higher PAI-1 levels in MI survivors and these levels were correlated with the recurrence of MI [136].

Lupu F. et al. studied the presence of t-PA and u-PA in sections of human aortas with atherosclerotic lesions. This group demonstrated the presence of t-PA in thickened intima, the media, and over luminal endothelial cells. They also detected a signal for t-PA in smooth muscle cells located both in intima and in the media, in foam cells positive for macrophage-specific markers and neo-microvessels located in the fibrous cap of the plaque. Besides the cellular localization, they detected extracellular t-PA in association with fibrin deposits in advanced atherosclerotic plaques. The analysis of u-PA shows its presence in macrophage rich intima and in SMC-rich areas. Moreover, the study showed large amounts of u-PA in the areas of the plaque shoulders and core [137].

Another study conducted by Salame et al. aimed to analyse the expression of tPA, uPA, and PAI-1 in normal and atheromatous vascular tissues obtained during coronary and peripheral vascular surgery. They used immunohistochemistry to localize tPA, uPA, PAI-1, and an immunoactivity assay to quantify their activities. The group also used QRT-PCR and in situ hybridization to quantify and to localize tPA, uPA, and PAI-1 mRNA. Their results showed that in a normal artery the expression of tPA, uPA, and PAI is low and is associated with the endothelium and with intimal and medial SMC. Compared with normal arterial tissue, in complex atheroma tPA, uPA, and PAI-1 were associated with endothelial cells, SMC, and areas rich in macrophages. In complex atheroma, PAI-1 mRNA expression associated with SMC was greatly increased but, interestingly, immunoactivity assays showed a low activity of PAI-1 compared with a normal artery [138].

It seems that in fibroproliferative lesions the expression of PAI-1 is low compared to a substantial amount of PAI-1 in complex atheromatous lesions. An explanation would be the presence of an intense inflammatory process in complex atheromatous lesions. Even if in the atheromatous plaques the amount of PAI-1 is very high, it seems that the balance is directed towards fibrinolysis. It is claimed that although the amount of PAI-1 is increased, its activity at the level of the plaque is reduced because it would be mostly inactive [138].

The idea that the activation of plasminogen leads in turn to the activation of a chain of metalloproteinases was issued. The level of these proteases can be measured by ELISA, and there are some studies that support the involvement of metalloproteinases (MMP-1, MMP-13, and MMP-8) from the collagenase family in atherosclerotic plaque development, progression, and instability [139]. This activation leads to the degradation of the matrix that makes possible the action of proliferation and migration stimuli on the SMC [139]. There is a recent study that associated a high level of MMP-7 with a high risk for atherosclerotic cardiovascular disease in adult patients with hypertrophy and diastolic dysfunction [140].

### 4.3. Von Willebrand Factor (VWF)

Another component that has been associated with atherosclerosis is the von Willebrand factor. Even though its major role is to participate in platelet adhesion, aggregation [141], and thrombus formation, von Willebrand factor apparently also has a role in the development of atherosclerotic lesion [142]. This glycoprotein is normally found in plasma, platelet alpha granules, endothelial cells (mainly in Weibel-Palade bodies), and the subendothelium [143].

Endothelial cells secrete von Willebrand factor that self-associates. A part of these multimers remains at one end associated at the surface of the endothelial cell; the other end stretches over a length of 100 µm in the direction of blood flow [144]. Stress conditions on the vascular wall cause the unfolding of VWF and exposure of some sites of interaction with platelets, but also exposure for self-association; a structure with a beads on a string appearance is formed and this may play an important role in VWF–platelet thrombi formation [145]. It was shown in a murine model that an increased VWF-mediated platelet adhesion to the endothelium characterizes the early stage of atherosclerosis [146].

A factor that mitigates the self-association of VWF is the apoA-I (apolipoprotein AI) contained in HDL. It is assumed that in inflammation the level of HDL and apoA-I decreases and there is a change in the composition of this lipoprotein, when apoA-I is replaced by amyloid-A [147,148]. On the other hand, the level of LDL increases in inflammation, together with VWF self-association. LDL-c is cytotoxic to endothelial cells and this damage is an important early step in atherosclerosis. These findings would explain why the high LDL/HDL ratio found in inflammation participates in promoting atherosclerosis [149,150].

There are certain observations that support the involvement of VWF in the formation of atherosclerotic plaque such as: increasing the number of Weibel–Palade bodies at the level of atherosclerotic plaque [151], inducing Weibel–Palade body exocytosis by oxidized low-density lipoprotein [152], and increasing VWF expression at the level of arterial bifurcations, areas where atheroma plaques develop [153].

There are numerous studies that associate a high level of VWF and an increased risk of cardiovascular disease [154]. Two studies conducted by Sannoveld et al. showed that the coronary plaque burden of unstable angina pectoris was associated with high VWF levels, whereas high risk coronary lesions were not associated with VWF levels. On the other hand, they found larger values of VWF antigen in acute coronary syndrome than in stable angina pectoris (1.73 IU/mL vs. 1.26 IU/mL). These values were obtained using ELISA with polyclonal Rabbit anti-Human VWF. Independent of plaque burden, high VWF levels are predictive of adverse cardiovascular outcome and death during one-year follow-up in acute coronary syndrome and stable angina pectoris patients [154,155].

Even though a pathogenetic role of VWF has been suggested, mainly by thrombus formation, studies with type 3 von Willebrand disease failed to show any atherosclerotic lesion reduction [156,157]. Therefore, although an increased level of VWF is associated with a high risk of coronary artery disease, the exact mechanism is not clear and further studies are necessary.

## 5. Conclusions

Atheroma development has severe and potentially lethal consequences. Given the particular dynamics of the disease characterized by a long interval of clinical silence, followed by a sudden event, we stress the importance of developing diagnostic methods and markers particularly tailored to detect and predict the evolution of incipient vascular lesions.

We have identified a few biomarkers that show potential for such a role and for which copious relevant data are available. The role of well-known lipid metabolism markers such as serum LDL-c, HDL-c, and triglycerides levels in clinical management of dyslipidaemia is well understood. New lipoprotein markers such as sdLDL (which pairs lower affinity to LDL receptor and increased penetrability into the subendothelial space) and Lp (a) could be used to guide therapy and improve outcomes. The reported involvement of ceramides in the pathogenesis of atherosclerosis by promoting LDL infiltration into the vascular wall and by promoting endothelial lesion by reducing nitric oxide production may indicate a potential marker role. Even though these biomarkers are not yet included in standard clinical practice, diagnostic tests for circulating ceramides are already available in some clinics. Therefore, there is a strong and sustained initiative in place that strengthens their role in evaluating atherosclerosis.

Apart from serum lipid-related compounds, inflammatory markers were associated to the atheroma formation process. hs-CRP, a low-grade inflammation biomarker, is already included in standard clinical practice to stratify the cardiovascular risk. The plasminogen activator (PA) system and its inhibitor PAI-1 play a putative role in the pathogenesis of atheroma formation by intimal mechanism such as promoting cell migration and modulating the turnover of fibrin and extracellular matrix. A potential atheroma biomarker role may be considered for such molecules.

Accumulating evidence has shown that metalloproteinases may be used as biomarkers in secondary prevention, since they can help to predict patients with high atherosclerotic risk. MMP-2, MMP-8, and MMP-9 are considered representative molecules that can assess plaque vulnerability and, therefore, more precisely predict the stage of the disease. In addition, PTX3 is considered a useful biomarker for disease severity and outcome. Nevertheless, further studies need to be conducted to establish specific cut-off points for MMP and PTX3 that characterize different stages of the disease.

The complexity of the atherosclerotic process and the plethora of molecules involved make assessing the cardiovascular risk a difficult task. Furthermore, the population diversity characterized by a variety of genotypes, phenotypes, and gender differences, together with cultural and lifestyle variability, also contributes to the complexity of a hypothetical comprehensive model of atheroma formation and evolution.

Although considerable advances are reported, such a model does not yet exist, and the ideal biomarker has not been identified. Perhaps composite multiplexed instruments grouping different available biochemical parameters could be useful to improve the diagnosis, staging, and monitoring of the atherosclerotic processes.

## Figures and Tables

**Table 1:** Components of CERT1 and CERT2 risk scores (adapted after [60]).

CERT1	CERT2
Cer(16:0)	Cer(24:1)/Cer(24:0)
Cer(18:0)	Cer(16:0)/PC(16:0/22:5)
Cer(24:1)	Cer(18:0)/PC(14:0/22:6)
Cer(16:0)/Cer(24:0)	PC(16:0/16:0)
Cer(18:0)/Cer(24:0)	
Cer(24:1)/Cer(24:0)	

Note: Cer, ceramide; PC, phosphatidyl choline.

## Data Availability

Not applicable.
